# Perception of first respiratory infection with *Pseudomonas aeruginosa* by people with cystic fibrosis and those close to them: an online qualitative study

**DOI:** 10.1136/bmjopen-2016-012303

**Published:** 2016-12-26

**Authors:** Sally C Palser, Oliver C Rayner, Paul A Leighton, Alan R Smyth

**Affiliations:** 1 Division of Child Health, Obstetrics and Gynaecology, School of Medicine, University of Nottingham, Nottingham, UK; 2 Person with Cystic Fibrosis; 3 NIHR Research Design Service for the East Midlands, School of Medicine, University of Nottingham, Nottingham, UK

**Keywords:** RESPIRATORY MEDICINE (see Thoracic Medicine)

## Abstract

**Background:**

People with cystic fibrosis (CF) are susceptible to respiratory infection with *Pseudomonas aeruginosa* (PA), which may become chronic if initial eradication fails. Environmental acquisition and person-to-person transmission can occur. Respiratory PA infection is associated with increased mortality and more hospitalisations. This may cause patients and families anxiety and lead them to adopt preventive measures which may be ineffectual and intrusive. It is not possible to hold a conventional focus group to explore these issues because people with CF cannot meet together due to the risk of cross-infection.

**Objective:**

To explore the perceptions of first respiratory infection with PA in people with CF and those close to them.

**Design:**

We designed an online survey, to maximise accessibility and avoid the risk of cross-infection. This established the respondent's relationship with CF, asked 3 open questions about perceptions of PA and a final question about the prioritisation of research. Responses were analysed using a structured, iterative process. We identified keywords, analysed these incontext and derived key themes.

**Setting:**

Promotion through social media allowed respondents from any country to participate.

**Participants:**

People with CF and those close to them.

**Results:**

Responses were received from 393 people, including 266 parents and 97 people with CF. The key themes were the emotional burden of PA (fear in particular); the burden of treatment PA entails and the need for accurate knowledge about PA.

**Conclusions:**

Lack of knowledge and the health beliefs of individuals may promote fear of infection and inappropriate avoidance measures. Uncertainty about the implications of PA infection and the treatment required may cause anxiety. Healthcare professionals should provide clear information about how PA might be acquired and the treatment necessary, making clear the limitations of current understanding and acknowledging health beliefs.

**Strengths and limitations of this study**

- This is a large qualitative study which gives new insights into the views of people with cystic fibrosis (CF) and those close to them, on the first respiratory infection with *Pseudomonas aeruginosa*.
- Our online survey, promoted through social media, allowed wide participation and avoided the risks of cross-infection.
- Our novel methodology, allowed analysis of responses to open questions, leading to key ‘themes’ identified by participants, which should help clinicians communicate more effectively with patients and families.
- This was an online survey and so we were unable to ask further questions to explore the views of participants in depth.
- To encourage the maximum number of responses, we did not collect information about the respondent apart from their relationship with CF.

## Introduction

*Pseudomonas aeruginosa* (PA) infection is arguably the most important determinant of survival in people with cystic fibrosis (CF). Chronic respiratory infection with PA drives the cycle of infection, inflammation and lung damage, causing accelerated lung function decline,^1^ more frequent exacerbations,^2^ increased treatment burden and ultimately accelerated mortality.^1^

Early respiratory infection with PA can be eradicated by a number of antibiotic regimens,^3^ but these all require a significant commitment from patients and families. If not eradicated, chronic PA management requires regular inhaled antibiotics and necessitates an even greater, life-long, treatment burden.^4^

PA is an environmental organism, widely found in soil and water. Unfortunately, neither the specific source of the infecting organism nor the mechanism by which infection occurs are well understood. Cohort segregation by microbiological status was adopted in Denmark in the 1980s in an attempt to prevent new infection^5^ and has since been implemented worldwide. While this may reduce acquisition through cross-infection in the CF centre, respiratory PA infection may be acquired from the home environment. However, research has shown that in only a minority of patients is the newly infecting PA strain genetically identical to one found in the patient's home.^6^ It is also possible that an infected patient contaminates the home environment, having acquired infection elsewhere. There is little information about acquisition of respiratory PA infection in the home environment designed to help people with CF (or parents of affected children) make decisions about sensible preventive measures. In the absence of clear information and advice, there is evidence that parents of children with CF may be very anxious about PA acquisition and take a variety of measures in an attempt to prevent infection, which may be intrusive.^7^ There are little published data on the views of adults with CF.

Our objective was to understand attitudes towards first respiratory infection with PA among people with CF, the parents of affected children, extended families and close friends. A better understanding of these attitudes could help healthcare professionals communicate information about respiratory infection with PA more effectively.

## Methods

### Data collection

**Figure 1:**
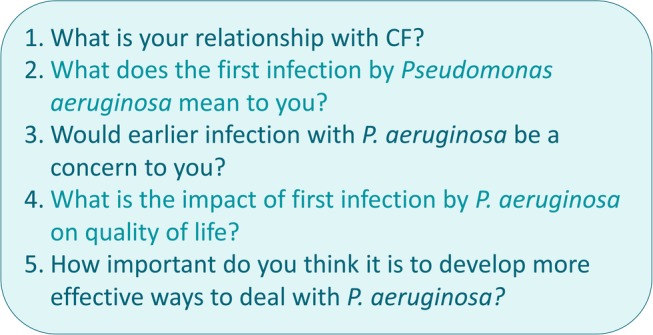
Five-question SurveyMonkey survey.

We aimed to investigate the beliefs and perceptions of people with CF, and those close to them, about respiratory infection with PA. We designed a five-question survey, based on the experiences of people with CF (see figure 1). We used the SurveyMonkey online survey tool (http://www.surveymonkey.com) to collect responses. The survey allowed narrative responses of any length and consisted primarily of open-response questions. The survey link was distributed through personal Facebook and Twitter accounts; the UK Cystic Fibrosis Trust (http://www.cysticfibrosis.org.uk); the CF Aware social media channel (#cfaware) and through CF groups on Facebook. People completing the survey were invited to share the link with other interested parties. The survey was open for responses between 6 and 18 May 2014.

Data were captured automatically by the SurveyMonkey web service and exported for data handling and analysis to the N'Vivo qualitative data software package (QSR International Pty, 2014).

The Research Ethics Committee of the Faculty of Medicine and Health Sciences, University of Nottingham, indicated that the research did not require formal ethical approval.

### Data analysis

To reflect the digital nature of the data (i.e., collected online) and the potential for variation in the quality and quantity of data (ie, respondents may enter as little or much as they choose), a novel structured, iterative approach to data analysis was adopted. Keyword (summative content) analysis^8^ and keyword-in-context-analysis^9^ were used to support a broad understanding of the data set, and a more conventional qualitative, thematic analysis used to illustrate broad trends and interesting features in the data. Analysis was iterative insomuch that keyword analysis informed those words incorporated into the keyword-in-context-analysis and the keyword-in-context-analysis informed the thematic analysis.

### Keyword analysis

Using the NVivo ‘word frequency’ function, word counts were performed for each open-response question. Words of the same derivation (scare, scary, scared, etc) were linked and short words (3 characters or less) were excluded. The 25 most frequently used words for each question were recorded.

### Keywords-in-context

Keyword counts were reviewed by the authors to inform the keywords-in-context analysis. Ten words from each list of 25 were selected; selection was informed by word count frequency and by a subjective assessment of word significance, for example, frequently used words such as *lung, think, feel* and *infection* might be excluded at this point as they offer little substantive insight (ie, they may simply introduce or link a comment). We made adjustments where words were preceded by a modifier word (eg, ‘scary’ preceded by ‘not’). Other factors such as emotive words or words used by one population group in isolation might lead to less frequently used words being included at this stage. Keywords-in-context were selected independently by two authors (SCP and PAL) with disagreements adjudicated by a third author (ARS). Final lists were agreed by all authors, representing a range of perspectives on the issue (clinical, personal, research).

The NVivo ‘text search’ and ‘word tree’ display functions were used to isolate selected keywords and demonstrate their usage. Summaries of word-usage for each keyword were generated, with a particular concern for consistency of use and notable usage.

### Thematic analysis

Keyword and keyword-in-context analyses may be taken here as part of the initial ‘analytic effort’ which constitutes a simple thematic analysis.^10^ Agreement on the interpretation of keywords-in-context supports the initial identification of ‘themes’ (broad areas of interest) and ‘codes’ (specific features, ideas, responses, etc) which can be applied to the whole data set.

The N'Vivo coding function was used to annotate data and collect instances where key themes and specific codes were manifest in participant responses. Coding was validated by two authors (SCP and PAL) and further discussed by all authors to ensure validity and appropriateness of interpretation.

Each data entry was considered independent of prior keyword analyses and each entry was considered in full and coded to as many themes/codes as appropriate. Organisation of coded data within the analytic themes presents a more clearly contextualised understanding of how PA is perceived and understood.

## Results

### Demographics

A total of 393 people responded to the survey—266 had 1 or more children with CF and 97 were people with CF. Extended family members accounted for 15 responses, 6 were partners/spouses, 5 respondents skipped the question and 2 were friends of individuals with CF. Two people had another relationship to CF; one was a healthcare worker and one had non-CF bronchiectasis. Included in the above were three people who identified to more than one category: two were people with CF who also had a sibling with CF; and one had CF and had a child with CF. The questionnaire did not ask if the respondent had direct experience of first infection with PA*.* However, in the free-text responses, 164 of 393 (42%) respondents made direct reference to their experience of first infection with PA*.* We did not collect data on respondent's age, gender or country of residence.

While most participants completed the survey in <5 min, some spent as long as 90 min completing it. The total data set includes 1955 distinct entries and in excess of 15 000 words.

### Keywords, ‘words in context’ and themes

**Figure 2:**
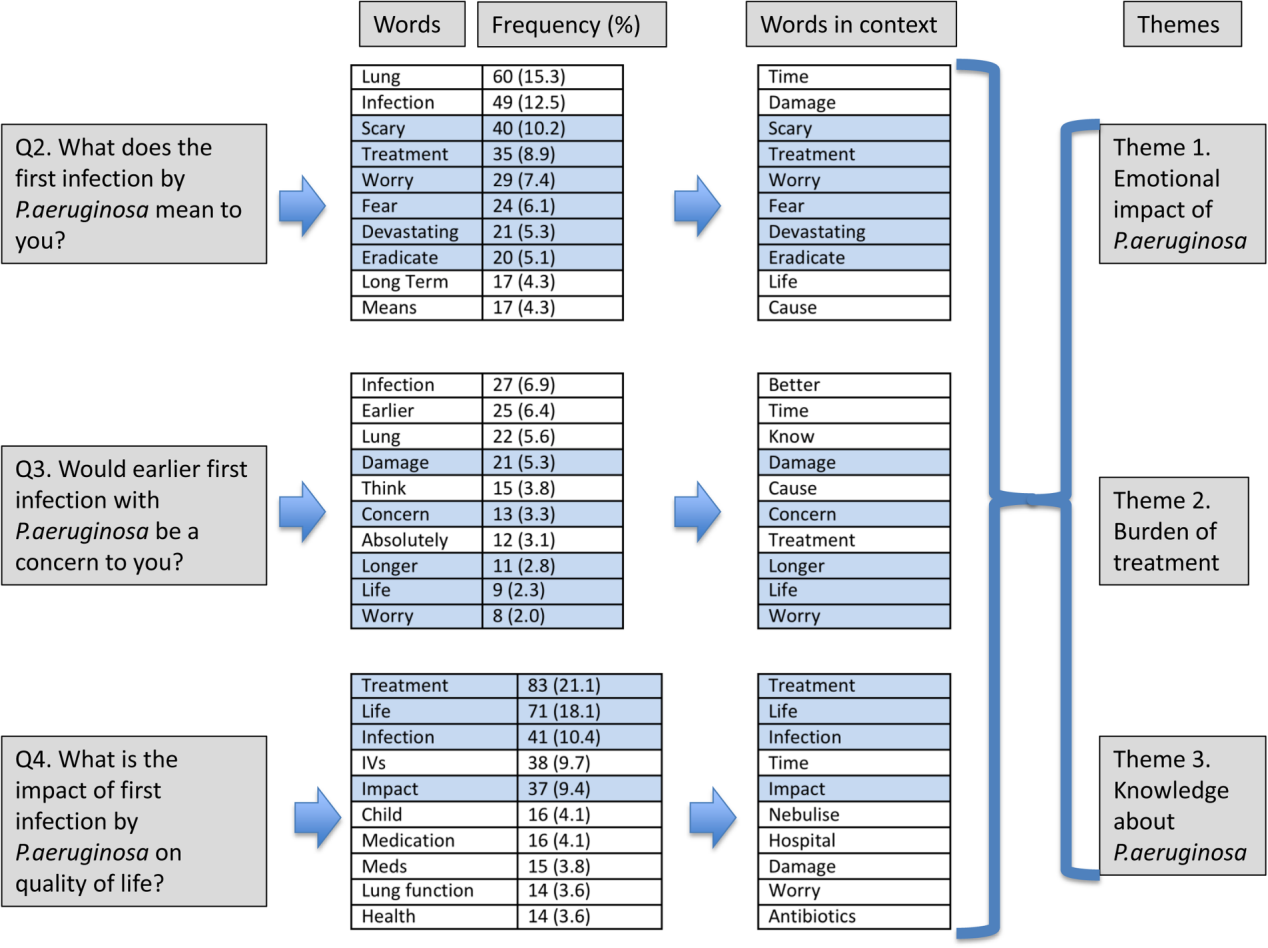
Flow diagram of qualitative analysis for research questions 2–4, showing progress through word frequency, ‘words in context’ and themes. Words retained from frequency analysis to ‘words in context’ are shaded in blue. The words in context (from questions 2–4) contributed to all of the three themes identified.

The results at each stage of the analysis, for questions 2–4, are summarised in figure 2. The questions are given in the three grey boxes on the left. The most commonly used words in response to each question are listed in the first column of tables (with the frequencies of each). The second column of tables lists the 10 most important words used in response to each question—selected from a list of 25 according to frequency and significance. The words retained from the frequency analysis to the ‘words in context’ selection are shaded in blue. The words in context (from questions 2 to 4) contributed to all of the three themes identified and these themes are shown in the grey boxes on the right.

**Figure 3:**
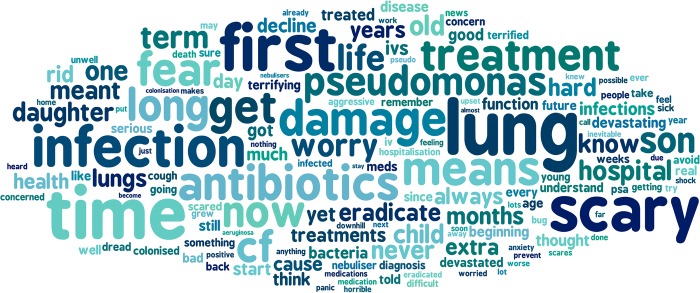
The most commonly used words in response to question 2.

The most frequently used words, from the first stage of the analysis (question 2), are represented in figure 3 as a ‘word cloud’.

One word would frequently be used in several contexts. For example, the word ‘time’ was used to mean:

- *A point in time*. ‘… the first time he got Pseudomonas!’
- *Time spent on treatment*. ‘More time spent on treatment…’
- *Time in relation to emotion*. ‘Scary time as not sure what it all meant’
- *Time meaning the future*. ‘…the earlier the infections start the more damage over time’.

Other words used in a variety of contexts were ‘know’ and ‘life’. From these analyses, three overarching themes were identified; the emotional impact of PA, the burden of treatment and knowledge about PA. Examples of the codes contributing to each theme are given in table 1, together with example quotations corresponding to each code.

**Table 1:** Summary of thematic analysis

Theme	Code	Example of data
Emotional impact	Fear	“My experience in relation to lung function was insignificant, however, the psychological effect was all-consuming. I even had to take time off work to get my head around it. The magnitude & significance was just too scary!” (patient)
	Resignation	“Terrifying bacteria we spend our lives trying to avoid, yet accept it is probably inevitable…” (parent)
	Realisation	“[pseudomonas is]…a slap in the face that CF is real” (parent)
Treatment burden	Restricting	“It means having to do more treatments every day, particularly nebulised antibiotics which are time consuming and restrict your freedom to travel and work. This also marks you out as being different from peers which can be hard to deal with psychologically” (patient)
	Stressful	“Now J needs nebs, our day is filled with resistance, arguing and tension because it's another thing he doesn't want to do…” (parent)
	Invasive	“The start of lots of extra powerful drugs that can cause lots of harm to the body of my son” (parent)
Knowledge	Lacking awareness	“Not something I had been pre-warned about or even read about” (parent)
	Incomplete	“I thought I was going to die very quickly after getting it” (patient)
	Beneficial	“…the earlier you know the harder you can hit it with treatment” (patient)

### Theme 1: emotional impact

The emotional impact of respiratory infection with PA on people with CF, their families and friends is considerable. The emotion respondents acknowledged most frequently was fear (152/393 (38.7%) of respondents reported fear or worry), ranging from simply ‘…scared…’ to specific fears, primarily of the negative health implications, “I get frightened about how much damage the infection will cause.” *Participant 118 (parent)*. Fear of recurrent infection remains after successful eradication, “…it's always in the back of your mind…are they going to grow it again?” *115 (parent)*. Ultimately, the fear is that PA acquisition will further shorten an already limited life, “A stage closer to death,” *388 (parent)*. For some, the fear itself is debilitating, “My experience in relation to lung function was insignificant, however, the psychological effect was all-consuming. I even had to take time off work to get my head around it. The magnitude & significance was just too scary!” *57 (patient).* The acquisition of PA may be a devastating blow—there is a sense of loss associated with the perceived change in health status, “…you think your child is invincible with mild CF until the first pseudomonas call.” *212 (parent)*.

Patients, and particularly parents, go to great lengths to avoid PA, “…we don't allow animals in our house, we don't visit friends’ houses with pets. We don't do horse riding. We are incredibly picky about where she uses swimming pools…” *3 (parent).* Acquisition is felt to be inevitable, but parents still feel that they must take active preventive measures, “Terrifying bacteria we spend our lives trying to avoid, yet accept it is probably inevitable…” *165 (parent)*. Measures taken to avoid PA may have a detrimental effect on development, particularly if it means a child missing out on school or time with friends, “…my son aged 7 is paranoid about it and misses out in so much activity for fear of getting it,” *111 (parent)*. For adolescents and adults, the health uncertainty affects their ability to make plans, “It means worrying about letting people down,” *367 (patient)*.

First infection with PA marks a turning point in people's relationship with CF. For many, it is, “…a slap in the face that CF is real.” *278 (parent).* Prior to PA infection people are more able to deny the seriousness of the condition. “I had always used it as a benchmark, i.e. ‘I'll be okay because I've never had Pseudo’,” *281 (patient)*. Acquisition therefore, causes a re-evaluation of general health and disease trajectory, psychologically marking a transition to ill-health.

### Theme 2: treatment burden

The additional treatment required after respiratory PA infection causes significant inconvenience to patients and their families. In particular, the introduction of extra nebulised treatment was commonly cited as a major impact of first infection, “…I now have an extra nebuliser or inhaler to do twice a day for the rest of my life—which means another hour of every day lost to treatments,” *163 (patient)*. Additional time spent on treatment conflicts with other activities for the person with CF which may worsen social isolation, “It means having to do more treatments every day, particularly nebulised antibiotics which are time consuming and restrict your freedom to travel and work. This also marks you out as being different from peers which can be hard to deal with psychologically,” 367 *(patient)*.

Many respondents mentioned the difficulties posed by more frequent hospital admissions, which followed the acquisition of PA. For the person with CF admission means absence from school or work, “…3–4 hospital admissions a year which has significantly affected my ability to attend school, uni and work,” *283 (patient)*. For parents, an admission similarly leads to time off work to support their child, “…rearranging work life etc. for hospitalization if required.” *195 (parent)*.

Nebulised drugs and hospital admissions are frequently novel treatment modalities after PA acquisition. For some families, the therapy itself is stressful, “Now J needs nebs, our day is filled with resistance, arguing and tension because it's another thing he doesn't want to do…” *113 (parent)*. Treatment may be more invasive, particularly where the patient requires an intravenous line—a procedure which in some cases requires a general anaesthetic, “He had to have I.V's for the first time and he was put under general anaesthetic.” *261 (parent)*. Some parents expressed concerns about the ‘strength’ of treatment needed after PA acquisition, particularly where their child was young, “The start of lots of extra powerful drugs that can cause lots of harm to the body of my son,” *217 (parent)*. Others reflected on side effects, such as skin photosensitivity with ciprofloxacin, “Being on cipro isn't so bad in winter but is tough in summer.” *324 (parent)*.

### Theme 3: knowledge

Knowledge about PA is beneficial and detrimental. Prior to respiratory infection with PA, many patients and parents are aware of the organism and the potential health consequences, and this knowledge may induce fear, “We panicked as only heard bad things about the infection,” *33 (parent)*. There is an assumption that PA infection will immediately lead to severe health decline or even death, “I thought I was going to die very quickly after getting it,” *16 (patient)*. Thus, prior, incomplete, understanding may worsen fear, “…even if you have successfully eradicated it, it is guaranteed you will get it again,” *156 (parent)*, and promote unrealistic avoidance measures, “…have turned into a clean freak to keep it away to the best of my ability…” *177 (parent)*.

Others have very little prior knowledge about PA, “Not something I had been pre-warned about or even read about,” *249 (parent).* Some perceived that there was insufficient support from the healthcare team, “We were left with no support…It was awful and desperately worrying until the next clinic appointment…” *235 (parent)*. Others, however, found that gaining greater understanding of the potential for eradication helped, “Was worried at first but I know it's treatable,” *242 (parent)*. The knowledge gained by experience of living with PA can be reassuring, “…10 years on I can look back and say it didn't cause the problems I thought it would,” *387 (parent).* Others found discovery of acquisition beneficial, “…the earlier you know the harder you can hit it with treatment,” *364 (patient)*.

Lack of clear advice about PA acquisition makes some parents restrict their child's activities, “…this has made me pretty paranoid about everything as pseudo is everywhere and whilst I know it shouldn't, as life is for living etc, it does make me stop my little boy from doing a few things due to the risks,” *151 (parent)*. Parents described replacing kitchens or bathrooms, or even moving house, due to concerns about the source of PA.

### Importance of PA for future research

Question 5 asked respondents to rate, on an ordinal scale, “How important do you think it is to develop more effective ways to deal with P. aeruginosa?” The scale ranged in five steps from ‘totally unimportant’ to ‘top priority/urgent’. The answer was given as ‘top priority/urgent’ by 314 of 393 (80.0%) respondents.

## Discussion

This study collected the views of a large number of people with CF, the parents of affected children, extended families and close friends, about first respiratory infection with PA. We used a novel web-based survey which was accessible to participants internationally. There was active promotion of the survey from within the CF community, since the survey author is a person with CF. We identified three prominent themes; emotional impact, treatment burden and knowledge. These themes should inform CF care teams, allowing them to provide more effective support for patients and those close to them.

Our study echoes the findings of previous work by Ullrich *et al*,^7^ who interviewed the parents of 21 children with CF about their attitudes to PA. They identified a spectrum of attitudes ranging from ‘bacterium-focused’ at one extreme to ‘child-focused’ at the other. Parents who were ‘bacterium-focused’ described taking measures to reduce their child's exposure to PA, both indoors and out, which could be restrictive and intrusive. A questionnaire survey from the same investigators^11^ described data from 130 parents from 10 German CF centres. Responses revealed that parents had incorrect beliefs about PA. They undertook an average of 11 ‘hygienic measures’, inside (eg, the affected child would use a different toothbrush morning and evening) and outside the home (the parents did not use air conditioning in the car). Parents felt stressed by the prospect of PA infection and the hygienic measures they believed would prevent this. Infection control guidelines in the USA^12^ give detailed advice on preventing cross-infection in the hospital setting but make few recommendations for infection control at home. European guidelines^13^ are restricted to infection control in CF centres. In the absence of specific guidelines, there is some evidence that physicians advocate a variety of preventive measures which may lead to confusion and uncertainty among people affected by CF.^14^

Previous qualitative work with adults with CF and parents of affected children has identified themes such as ‘from uncertainty to certainty’ and a desire for a ‘demanding yet normal life’.^15^ These themes relate closely to the themes of ‘knowledge’ and ‘treatment burden’ which we identified in our study. The theme of ‘treatment burden’ is also prominent in qualitative work, conducted with CF adults, who discussed their nebulised medication—an essential component both of PA eradication and long-term suppressive therapy for PA.^16^ This work describes ‘intentional non-adherence’ when adult CF patients must balance the demands of busy lives with the requirement for time-consuming therapy. Indepth interviews with CF adults have shown that many experience emotional symptoms such as frustration, sadness/depression, irritability, worry and difficulty sleeping.^17^ These symptoms are likely to be exacerbated by the anticipation or occurrence of significant life events, such as respiratory infection with PA. Relatives and others who are close to people with CF may adopt the role of ‘lay carers’, many of whom contributed to our qualitative survey. Previous qualitative work has shown that the information needs of ‘lay carers’ may be overlooked by CF teams.^18^

Qualitative researchers often use a semistructured interview or focus group methodology whereby indepth interviews are conducted with a small number of participants, until no new themes emerge (data saturation).^19^ These interviews allow supplementary questions (which are refined in successive interviews) to enrich the data collected. A focus group approach was not open to us because of the risks of cross-infection that would entail. While we were unable to develop and ask supplementary questions, our online format allowed us to include many more participants. Similarly, to encourage the maximum number of responses, we did not collect information about the respondent apart from their relationship with CF (eg country of residence and gender were not collected). The large number of respondents increased the reach of our research and the generalisability of our findings.

It is not yet possible for CF teams to provide certainty about the risks for acquisition of PA, without considerable further research. Such research was strongly supported by respondents to our survey. CF teams should support patients (and those close to them) in addressing fear of PA acquisition, through proposing only those preventive measures supported by good evidence and by acknowledging the health beliefs of patients and families. Further research should also explore the information needs of people with CF and compare these with the needs to parents, so that information can be tailored appropriately. Our data indicate that first acquisition of PA marks a turning point in the relationship with CF for many patients and parents. CF teams should therefore ensure that sufficient time is available for discussion, when a new infection occurs, as well as providing clear information about treatment and the likelihood of successful eradication.
